# Time‐trends and treatment gaps in the antithrombotic management of patients with atrial fibrillation after percutaneous coronary intervention: Insights from the CHUM AF‐STENT Registry

**DOI:** 10.1002/clc.23316

**Published:** 2019-12-18

**Authors:** Laurie‐Anne Boivin‐Proulx, Ariane Deneault‐Marchand, Alexis Matteau, Samer Mansour, François Gobeil, John A. Camm, Keith A. A. Fox, Brian J. Potter

**Affiliations:** ^1^ Centre hospitalier de l'Université de Montréal (CHUM) Research Center and Cardiovascular Center Montreal Canada; ^2^ St. George's University of London London UK; ^3^ Centre for Cardiovascular Science and Royal Infirmary Edinburgh UK

**Keywords:** acute coronary care, antiplatelet therapy, percutaneous coronary intervention, atrial fibrillation

## Abstract

**Background:**

The management of atrial fibrillation and flutter (AF) patients undergoing percutaneous coronary intervention (PCI) has undergone a rapid recent evolution. In 2016, the Canadian Cardiovascular Society (CCS) published expert recommendations to help guide clinicians in balancing bleeding and thrombotic risks in these patients.

**Hypothesis:**

Antithrombotic regimen prescriptions for AF patients undergoing PCI evolved after the publication of the 2016 CCS AF guidelines.

**Methods:**

A prospective cohort of AF patients undergoing PCI with placement of a coronary stent from a single tertiary academic center was analyzed for the recommended antithrombotic regimen at discharge. Prescribing behavior was compared between three time periods (Cohort A [2010‐2011]; Cohort B [2014‐2015]; Cohort C [2017]) using the *χ* ^2^ test. In addition, antithrombotic management in Cohorts B and C were compared to guideline‐recommended therapy.

**Results:**

A total of 459 patients with AF undergoing PCI were identified. Clinical and procedural characteristics were similar between cohorts, with the exception of an increase in drug‐eluting stent (DES) use over time (*P* < .01). Overall, the rate of oral anticoagulation (OAC) increased over time (*P* < .01), associated with an increase in nonvitamin K OAC prescription (*P* < .01) and a concomitant decrease in vitamin K antagonist prescription (*P* < .01). Despite this, the overall rate of anticoagulation remains below what would be predicted with perfect guideline compliance (75% vs 94%, *P* < .01).

**Conclusion:**

There has been a dramatic shift in clinical practice for AF patients requiring PCI, with increases in prescription of OAC even in the context of an increase in the use of DES. However, room for further practice optimization still exists.

## 1 INTRODUCTION

Contemporary antithrombotic management of patients with either atrial fibrillation/flutter (AF) or coronary artery disease (CAD) has largely been well defined in clinical guidelines.^1^, ^2^, ^3^, ^4^ However, up to 30% of patients with AF also have CAD^5^ and the optimal management of AF patients requiring percutaneous coronary intervention (PCI) has, up until recently, been less clear. While oral anticoagulation (OAC) is indicated for the prevention of stroke and systemic embolism in most cases of AF,^6^ dual antiplatelet therapy (DAPT) is recommended after PCI in patients without AF.^7^, ^8^ Simply combining these two recommendations in patients with AF requiring PCI (so‐called triple antithrombotic therapy, TATT) increases the bleeding risk significantly.^9^

In 2016, both the Canadian Cardiovascular Society (CCS) and European Society of Cardiology (ESC) published expert recommendations to help guide clinicians in balancing bleeding and thrombotic risks in these patients.^1^, ^3^ The landmark PIONEER AF‐PCI^10^ was also published in 2016, followed closely by REDUAL^11^ and then AUGUSTUS,^12^ providing further evidence in support of nonvitamin K oral anticoagulation (NOAC)‐based antithrombotic regimens that could minimize the bleeding risk in AF patients having benefitted from PCI.

A recent international multicenter analysis demonstrated that the availability of newer antiplatelet and anticoagulant agents was associated with a significant increase in practice variability in the management of AF patients post‐PCI, but also that a major shift in clinical practice would be necessary in order to align with AF guidelines.^13^ We therefore sought in this analysis to determine whether the publication of the 2016 CCS and ESC guidelines, in conjunction with landmark clinical trials, were associated with such a change in practice patterns and to assess the size any residual treatment gap.

## 2 METHODS

We conducted a single‐center retrospective cohort analysis of a prospective registry in accordance with the Strengthening the Reporting of Observational Studies in Epidemiology guidelines.^14^ The need for informed consent was waived by the local institutional research committee. The study protocol was consistent with the ethical guidelines of the 1975 Declaration of Helsinki as testified by the approval of the institution's research committee. All consecutive AF patients >18 years of age undergoing PCI with coronary stenting at the Centre hospitalier de l'Université de Montréal during three time periods of interest were enrolled^1^: Cohort A, representing a “historic” period prior to the availability of newer P2Y12‐inhbitors and NOACs (January 2010 to December 2011)^2^; Cohort B, corresponding to a “pre‐guidelines” period (January 2013 to December 2015) where novel antithrombotics were clinically available, but evidence‐based guidance for this patient population was lacking; and Cohort C, a “post‐guidelines” period (January to December 2017). Patients with additional non‐AF indications for, or a documented contraindication to OAC, were excluded from the analysis. Additionally, four patients, who participated in a clinical trial and for whom the type of antithrombotic therapy could not be determined, were excluded.

The primary outcome of interest was the antithrombotic (antiplatelet and anticoagulation) regimen recommended at hospital discharge. Data regarding baseline patient characteristics, clinical presentation, procedural data, and in‐hospital outcomes were also extracted from hospital medical records.

Baseline characteristics of patients and procedural data are presented both in aggregate and separately for the three cohorts. Continuous data are expressed as mean with SD, and categorical/binary data are expressed as counts and percent proportions. Baseline comparisons between Cohort B and Cohort C were made using a one‐way ANOVA or the Kruskal‐Wallis test, as appropriate, for continuous data, the median test for ordinal data, and the *χ* ^2^ test for categorical data. The primary analysis consisted of an evaluation of the difference in prescription patterns across cohorts using the *χ* ^2^ test. Secondarily, we performed an evaluation of the differences between antithrombotic prescription patterns in the pre‐ (Cohort B) and postguidelines (Cohort C) cohorts and the patterns that would have been expected in those cohorts according to the 2016 CCS AF Guidelines.

The expected treatment with perfect guideline adherence was determined by first assessing the indication for anticoagulation for each patient by calculating each individual's CHADS_2_ score and combining it with consideration of the patient's age (≥65 years), as recommended in the 2016 AF guidelines. Patients with an estimated glomerular filtration rate (eGFR) ≥30 mL/min (Cockroft‐Gault formula)^15^ and a CCS guidelines indication for OAC would be expected to receive a NOAC‐based regimen, whereas those with eGFR <30 mL/min would receive VKA, in accordance with monograph recommendations for most NOACs in Canada at the time (apixaban had an indication for eGFR as low as 25 mL/min). Patients without a guideline indication for oral anticoagulant (OAC) were expected to receive DAPT at discharge. These expected treatments were then compared to the observed treatments in Cohorts B and C using the *χ* ^2^ test.

All statistical analyses were conducted using SAS 9.3 statistical software (SAS Institute, Cary, North Carolina). A two‐tailed *P*‐value <.05 was considered statistically significant without correction for multiple analyses.

## 3 RESULTS

A total of 459 patients with AF undergoing PCI were included across all three cohorts. Clinical and procedural characteristics of patients in the Cohort A (n = 109), Cohort B (n = 246), and Cohort C (n = 104) are detailed in Table 1 and were by and large similar between cohorts, with the exception of an increase in the use of drug‐eluted stent (DES) over time (37% vs 61% vs 94%, *P* < .01). The in‐hospital mortality rate was 3% overall, and in‐hospital major bleeding was 2% (Table 1).

**Table 1:** Characteristics and antithrombotic management of AF patients post‐PCI

	Total cohort	Cohort A (2010‐2011)	Cohort B (2014‐2015)	Cohort C (2017)	*P*‐value^a^
Baseline Characteristics	N = 459	N = 109	N = 246	N = 104	
Age, y ± SD	73.2 ± 9.4	72.3 ± 9.3	73.0 ± 9.5	74.4 ± 9.0	.23
Male sex, n (%)	333 (73%)	81 (74%)	177 (72%)	75 (72%)	.89
Diabetes, n (%)	198 (43%)	41 (38%)	104 (42%)	53 (51%)	.13
Hypertension n (%)	326 (71%)	68 (62%)	173 (70%)	85 (82%)	<.01
Stroke, n (%)	38 (8%)	14 (13%)	17 (7%)	7 (7%)	.14
Heart failure, n (%)	116 (25%)	24 (22%)	68 (28%)	24 (23%)	.45
Bleeding history, n (%)	16 (3%)	1 (1%)	8 (3%)	7 (7%)	.07
Body mass index, kg/m^2^ ± SD	27.8 ± 6.1	27.5 ± 5.7	28.1 ± 6.3	27.7 ± 6.2	.66
eGFR, mL/min ± SD	69.5 ± 35.8	69.1 ± 38.5	70.5 ± 36.6	67.5 ± 30.7	.77
CHADS_2_, median (IQR)	2 (1‐3)	2 (1‐3)	2 (1‐3)	2 (1‐3)	.22
HASBLED, median (IQR)	2 (1‐3)	2 (2‐3)	2 (2‐3)	1 (1‐2)	<.01
DES use, n (%)	287 (63%)	40 (37%)	150 (61%)	98 (94%)	<.01
ACS, n (%)	369 (80%)	98 (90%)	211 (86%)	60 (58%)	<.01
Admission medication	N = 459	N = 109	N = 246	N = 104	
Antiplatelet therapy					
ASA, n (%)	307 (67%)	83 (76%)	169 (69%)	55 (53%)	<.01
P2Y12, n (%)	48 (11%)	5 (5%)	29 (12%)	14 (13%)	.06
Clopidogrel, n (%)	39 (9%)	4 (4%)	23 (9%)	12 (12%)	.87^b^
Prasugrel, n (%)	1 (0%)	1 (1%)	0 (0%)	0 (0%)	
Ticagrelor, n (%)	8 (2%)	0 (0%)	6 (2,4%)	3 (2,7%)	
Anticoagulation					
OAC, n (%)	276 (60%)	50 (46%)	155 (63%)	71 (68%)	<.01
VKA, n (%)	135 (30%)	45 (41%)	75 (30%)	15 (14%)	<.01
NOAC, n (%)	141 (31%)	5 (5%)	80 (33%)	56 (54%)	<.01
In‐hospital events	N = 459	N = 109	N = 246	N = 104	
Major bleeding (BARC 3 or 5)	10 (2%)	1 (1%)	5 (2%)	4 (4%)	.28
Death	12 (3%)	5 (5%)	3 (1%)	4 (4%)	.13
Discharge medication	N = 447	N = 104	N = 243	N = 100	
Antiplatelet therapy					
ASA, n (%)	436 (98%)	104 (100%)	242 (100%)	90 (90%)	<.01
P2Y12, n (%)	441 (99%)	104 (100%)	243 (100%)	94 (94%)	<.01
Clopidogrel, n (%)	402 (91%)	104 (100%)	212 (87%)	86 (86%)	<.01^b^
Prasugrel, n (%)	3 (1%)	0 (0%)	2 (1%)	1 (1%)	
Ticagrelor, n (%)	36 (8%)	0 (0%)	29 (12%)	7 (7%)	
Anticoagulation					
OAC, n (%)	193 (43%)	34 (33%)	84 (35%)	75 (75%)	<.01
VKA, n (%)	107 (24%)	34 (33%)	61 (25%)	12 (12%)	<.01^b^
NOAC, n (%)	86 (19%)	0 (0%)	23 (9%)	63 (63%)	
Combination therapy					
DAPT, n (%)	252 (56%)	70 (67%)	159 (65%)	23 (23%)	<.01^b^
TATT, n (%)	181 (40%)	34 (33%)	83 (34%)	64 (64%)	
Dual pathway, n (%)^b^ (OAC + P2Y12)	8 (2%)	0 (0%)	1 (0%)	8 (8%)	

Abbreviations: ACS, acute coronary syndrome; ASA, acetylsalicylic acid; DAPT, dual antiplatelet therapy; DES, drug‐eluting stent; eGFR, estimated glomerular filtration rate; NOAC, nonvitamin K oral anticoagulant; TATT, triple antithrombotic therapy; OAC, oral anticoagulant; VKA, vitamin K antagonist.

a Significance applies to difference between Cohorts B and C only.

b *P*‐value for the distribution of OAC or P2Y12‐inhibitor type at baseline and on discharge or of the distribution of combination therapy. Novel P2Y12‐inhibitors prasugrel and ticagrelor were grouped together, as were OAC‐based regimens, to avoid cells with a zero count.

Antithrombotic prescriptions at both admission and discharge in each cohort are shown in Table 1. There was a significant increase in baseline use of OAC between the pre‐ and postguidelines cohorts (*P* < .01) despite a decrease in VKA use (*P* < .01) due to a marked rise in NOAC use over time (*P* < .01). A significant increase in P2Y12 inhibitor use at baseline was also observed (*P* < .01).

Discharge antithrombotic prescriptions also evolved significantly over time. The rate of OAC use at discharge was significantly higher in Cohort C compared to the preguidelines cohorts (*P* < .01), driven by a significant increase in use of NOAC (*P* < .01) at the expense of postprocedure VKA prescription (*P* < .01). Consequently, TATT prescription increased significantly (*P* < .01), whereas DAPT prescription at discharge was reduced (*P* < .01). The emergence of a dual pathway (anticoagulant plus a P2Y12‐inhibitor) prescription pattern was also observed in the most recent (postguidelines) cohort (Cohort C).

“Real‐world” and corresponding theoretical CCS guideline‐recommended OAC rates (based on the patient characteristics in each of Cohorts B and C) are presented in Table 2. Since the publication of the CCS guidelines in 2016, a clear change in clinical practice was observed, with a significant increase in the rate of anticoagulation following PCI from 35% to 75% (*P* < .01) and the rate of NOAC prescription at discharge increasing from 23% to 63% (*P* < .01) after the publications of the 2016 CCS AF guidelines. Despite this, the overall rate of anticoagulation (75%) and of NOAC prescription (84% of OAC) remains below what would be expected with perfect adherence with the 2016 CCS guidelines in the most recent postguidelines Cohort C (94% and 91%, respectively; *P* < .01 for both comparisons).

**Table 2:** Observed and guideline‐expected rates and type of oral anticoagulation in the pre‐ and postguidelines

Preguidelines	2014‐2015 observed (N = 243)	2016 CCS AF guidelines “Expected” (N = 243)	*P*‐value
Anticoagulation			<.01
No	159 (65%)	21 (9%)	
Yes	84 (35%)	222 (91%)	
Type of anticoagulant			<.01
NOAC	23 (27%)	199 (91%)	
VKA	61 (73%)	23 (9%)	
Postguidelines	2017 Observed (N = 100)	2016 CCS AF guidelines “Expected” (N = 100)	*P*‐value
Anticoagulation			<.01
No	25 (25%)	6 (6%)	
Yes	75 (75%)	94 (94%)	
Type of anticoagulant			<.01
NOAC	63 (84%)	86 (91%)	
VKA	12 (16%)	8 (9%)	

Abbreviations: NOAC, nonvitamin K antagonist oral anticoagulant; VKA, vitamin K antagonist.

## 4 DISCUSSION

This prospective registry of AF patients undergoing PCI with stent implantation highlights several findings relevant to clinical practice. First, the clinical characteristics of AF patients undergoing PCI have remained stable over time. Despite this, baseline P2Y12‐inhibitor and OAC use have increased and more patients are treated with NOAC at baseline than before. Discharge prescription of OAC has also significantly increased, due to substantial uptake of NOAC therapy, associated with an increased rate of TATT and dual‐pathway antithrombotic regimens. Also, despite this appropriate increase in intensity of antithrombotic management of AF patients in line with practice guidelines, operators at our institution have not avoided the use of DES in this population; a practice that now closely mirrors the treatment of non‐AF patients. Finally, despite these dramatic shifts in clinical practice, the overall rate of OAC prescription appears to remain somewhat below perfect guideline adherence, but clinically appropriate reasons for this discrepancy may not have been captured by our analysis.

The increased rate of NOAC prescription and a concomitant decreased VKA prescription reflects the impact of the CCS 2016 AF guidelines and landmark clinical trials.^3^, ^10^, ^11^ The higher TATT prescription rate is in agreement with the CCS 2016 AF guidelines recommendation of TATT for 3 to 6 months in these patients with CHADS2 score ≥ 2, placing greater weight on reduction of thromboembolic events and comparatively lesser weight on risk of major bleeding.^3^ A course of TATT of a duration of up to 6 months in patients at high risk of thrombosis was also advocated subsequently in the 2018 update of the CCS antiplatelet guidelines.^16^ The emergence of dual pathway antithrombotic therapy (anticoagulant plus a single antiplatelet agent) in clinical practice, on the other hand, represents an integration of randomized trial data from PIONEER AF‐PCI (rivaroxaban) and REDUAL (dabigatran) that showed that such a regimen could minimize bleeding risk without a signal for increase in clinical ischemic events.^10^, ^11^ A shift to dual pathway antithrombotic management is also advocated in the 2018 updates of the CCS antiplatelet and atrial fibrillation guidelines.^16^, ^17^ The recently published AUGUSTUS trial (apixaban), that also included medically managed ACS patients, also supports the safety advantage of dual pathway therapy over triple therapy.^12^ The ENTRUST‐PCI AF trial (edoxaban) furtherly reinforced the safety and anti‐ischemic efficacy of dual pathway regimens of dual pathway over triple therapy, with no significant difference in ischemic events between the two groups.^18^

Interventional cardiologists at our center no longer appear to be avoiding DES in AF patients when anticoagulation is indicated. Historically, bare‐metal stents (BMS) had been preferred for many patients requiring OAC because of the shorter DAPT duration required with BMS.^17^, ^19^ More recently, however, the recognition that shorter courses of DAPT (3‐6 months) with second generation DES are likely safe,^17^, ^20^, ^21^, ^22^, ^23^ combined with evidence of a safety advantage for NOAC‐based antithrombotic regimens is likely largely responsible for this observed change in stent choice. Additionally, recent studies among patients at high risk for bleeding, including those requiring OAC, have demonstrated the superior efficacy and safety of using certain DES platforms compared to BMS when shorter courses of DAPT are necessary.^24^, ^25^ Avoidance of restenosis with the use of DES may also help reduce the risk of bleeding complications by avoiding repeat procedures in a typically fragile AF population.^26^

## 5 LIMITATIONS

Certain limitations must be acknowledged given the retrospective nature of this analysis. First, while the registry is prospective and ongoing, data were abstracted from patients' medical records, giving rise to the possibility of ascertainment bias. Secondly, there is the potential for some “noise” around the ACS presentation variable due to the likely inclusion of some cases of crescendo angina as “unstable.” Nevertheless, we believe the impact of this variability to be minimal. Furthermore, the type of presentation (ACS vs non‐ACS) would not affect the recommended antithrombotic therapy prescribed at discharge according to the 2016 AF guidelines (though it would impact the duration). Finally, as this study was conducted in a single tertiary academic center, these results are not necessarily indicative of clinical practice in community centers or other Canadian academic centers.

## 6 CONCLUSION

While the impact of the availability of novel antithrombotic agents without clinical guidelines lead to increased practice variability, the combination of the 2016 CCS AF guidelines and landmark clinical trials appears to have had a major impact on antithrombotic regimen prescriptions for AF patients undergoing PCI at our center, with significantly higher rates of TATT and dual pathway regimen prescription in the most recent cohort. Guideline adherence was high overall, but room for improvement still exists, particularly in light of the most recent guidelines updates.

## CONFLICT OF INTEREST

The authors declare no potential conflict of interests.
